# To Preserve Ice

**Published:** 1870-07

**Authors:** 


### TO PRESERVE ICE.

WHERE ice is scarce, yet necessary for the sick, it may be kept many hours by putting a lump in a pitcher or other vessel not much larger than itself, or by wrapping it up in a a piece of blanket and putting it between two feather pillows, tying them compactly together.

In quenching great thirst it is better to chew lumps of ice, than to drink ice-water, the latter gives a feeling of uncomfortable fullness, or a metallic taste.

In the thirst of dysentery, lumps of ice eaten freely not only quench the thirst, but have been known to cure the disease.

